# Comparing International Experiences With Electronic Health Records Among Emergency Medicine Physicians in the United States and Norway: Semistructured Interview Study

**DOI:** 10.2196/28762

**Published:** 2022-01-07

**Authors:** Gracie Garcia, Christopher Crenner

**Affiliations:** 1 Department of History and Philosophy of Medicine University of Kansas School of Medicine Kansas City, KS United States

**Keywords:** electronic health records, electronic medical records, health information technology, health information exchange, health policy, international, emergency medicine, medical informatics, meaningful use, burnout

## Abstract

**Background:**

The variability in physicians’ attitudes regarding electronic health records (EHRs) is widely recognized. Both human and technological factors contribute to user satisfaction. This exploratory study considers these variables by comparing emergency medicine physician experiences with EHRs in the United States and Norway.

**Objective:**

This study is unique as it aims to compare individual experiences with EHRs. It creates an opportunity to expand perspective, challenge the unknown, and explore how this technology affects clinicians globally. Research often highlights the challenge that health information technology has created for users: Are the negative consequences of this technology shared among countries? Does it affect medical practice? What determines user satisfaction? Can this be measured internationally? Do specific factors account for similarities or differences? This study begins by investigating these questions by comparing cohort experiences. Fundamental differences between nations will also be addressed.

**Methods:**

We used semistructured, participant-driven, in-depth interviews (N=12) for data collection in conjunction with ethnographic observations. The conversations were recorded and transcribed. Texts were then analyzed using NVivo software (QSR International) to develop codes for direct comparison among countries. Comprehensive understanding of the data required triangulation, specifically using thematic and interpretive phenomenological analysis. Narrative analysis ensured appropriate context of the NVivo (QSR International) query results.

**Results:**

Each interview resulted in mixed discussions regarding the benefits and disadvantages of EHRs. All the physicians recognized health care’s dependence on this technology. In Norway, physicians perceived more benefits compared with those based in the United States. Americans reported fewer benefits and disproportionally high disadvantages. Both cohorts believed that EHRs have increased user workload. However, this was mentioned 2.6 times more frequently by Americans (United States [n=40] vs Norway [n=15]). Financial influences regarding health information technology use were of great concern for American physicians but rarely mentioned among Norwegian physicians (United States [n=37] vs Norway [n=6]). Technology dysfunctions were the most common complaint from Norwegian physicians. Participants from each country noted increased frustration among older colleagues.

**Conclusions:**

Despite differences spanning geographical, organizational, and cultural boundaries, much is to be learned by comparing individual experiences. Both cohorts experienced EHR-related frustrations, although etiology differed. The overall number of complaints was significantly higher among American physicians. This study augments the idea that policy, regulation, and administration have compelling influence on user experience. Global EHR optimization requires additional investigation, and these results help to establish a foundation for future research.

## Introduction

### Background

Correlations between electronic health records (EHRs) and physician frustrations have been well described throughout informatics literature. The phenomenon of high user dissatisfaction is often attributed to increased administrative requirements, decreased face-to-face patient time, information overload, and limited interoperability [1-12]. This technology has been analyzed on both local and global scales [13-17]; however, few studies have compared users from different countries who practice in parallel clinical settings. Our study compares emergency medicine (EM) physician experiences with EHRs in the United States and Norway. In addition, societal and cultural differences are carefully considered while analyzing components that may affect user satisfaction.

Health information technology (HIT) is used in many countries, but deployment of EHRs vary [18-23]. Despite global HIT use, the United States is perhaps the most prominent generator of informatics research that emphasizes the shortcomings of this technology. Currently, there are few studies that consider or compare international EHR experiences. However, a recent study by Downing et al [24] found that even when using the same vendor (Epic Systems), American physicians had significantly longer documentation and were less likely to report satisfaction or improved work efficiency compared with those in Australia and Singapore. Our results contribute to this small body of research.

### Objectives and Measured Outcomes

This study considers factors that contribute to EHR user satisfaction by comparing individuals with similar professional responsibilities in different national contexts. Are the negative consequences of this technology shared among countries? Does it affect medical practice? What determines user satisfaction? Can this be measured between international cohorts? Are there specific factors that account for similarities or differences? This study explores these questions while considering influential variables from a sociopolitical–technological context.

Primary outcomes include the overall EHR experience and specific opinions within each cohort. This was achieved by conducting structured interviews in the hospital and observing behaviors within the physician’s typical working environment. Thematic analysis allowed the quantification and comparison of common topics. Notable differences may help to identify targeted solutions for HIT optimization. For example, if users from each cohort believe that software interfaces are challenging to use, it could indicate that technology-specific factors (understanding and using computers) significantly increase frustration. On the other hand, differences may identify solutions that may have otherwise been overlooked.

Secondary outcomes assess participant responses within a sociocultural context, as HIT infrastructure differs among countries [16-23,25]. Previous research shows that successful EHR use is greatly influenced by social and governmental constructs [9,24,26,27]. A general understanding of the current HIT status and health care infrastructure in the United States and Norway supports the interpretation of the data. We briefly discuss this information before proceeding.

### United States: Emergency Care and Current EHR Status

Since the Emergency Medical Treatment and Labor Act of 1986, hospitals must provide consultation, screening examination, ancillary testing, and stabilization of anyone concerned with a life-threatening condition, regardless of their ability to pay [28-30]. Patients are evaluated in the emergency department (ED) after arriving via ambulance, private vehicle, or walking in. Physicians who staff the ED receive formal EM training by completing an EM residency for 3-4 years following medical school [31]. Although the ED functions as a hospital’s gatekeeper, studies show that only a small number of ED visits result in admission [32]. National increase in low-acuity ED patient volume has been attributed to multiple factors including rising health care costs, primary care shortages, and lack of access to after-hour care [33-37].

Integration of technology and health care started in 2004 with the establishment of the Office of the National Coordinator for HIT, but widespread EHR use did not occur until after the HIT for Economic and Clinical Health Act was passed in 2009 [38]. This legislation provided monetary incentives for government-certified EHR adoption and implementation [39]. Pressure for rapid health care digitization generated numerous unintended consequences including industrial arms race that many policy makers did not consider [40-43]. As of 2017, 96% of hospitals in the United States had implemented technology certified by the Department of Health and Human Services [44].

The 21st Century Cures Act prohibits companies and organizations from intentionally restricting health information exchange (HIE) capabilities for monetary benefit [45]. Nevertheless, evidence indicates that *information blocking* still occurs in the United States [46,47], and a 2018 Report to Congress showed only 51% of hospital physicians had electronic access to necessary patient information from other facilities at the point of care [44]. The private sector has been meeting interoperability demands as evidenced by programs like Epic’s Care Everywhere [27]. However, the extent of clinical data availability is dependent on the participating facilities [48]. In 2018, the US Department of Veterans Affairs (VA) announced partnership with Cerner, an EHR company that will eventually be the sole vendor to all VA facilities that serve military populations [49].

### Norway: Emergency Care and Current EHR Status

Inpatient and specialist care are provided by state-owned hospitals and managed by 4 geographically distinct government subdivisions known as Regional Health Authorities [50,51]. A total of 428 local municipalities are responsible for supplying primary care including after-hour access [50]. Municipalities have urgent care centers with on-call physicians (*legevakt*) [50]. The ED or *acute receiving area* (*akuttmottak*) is only accessible via ambulance or physician referrals [50,52]. The department is traditionally staffed by internal medicine, neurology, orthopedics, and surgery physicians [53]; however, EM was recently recognized as an independent specialty in Norway in 2017 [52]. Historically, ambulance and other health personnel would communicate with hospitals to determine the most appropriate inpatient specialty service to receive the patient upon arrival [50].

Medical records from hospitals and outpatient facilities are not integrated, but messaging systems embedded within EHR software allow providers to collaborate [51]. In 2008, the government recognized the interoperability needs and launched a national HIE platform in 2012 known as *Core Journal* (*Kjernejournalen*) [54]. This gives all Norwegian physicians access to critical patient information, regardless of where previous treatment was provided [51,54]. It includes data necessary to prevent unfavorable outcomes that may be difficult to obtain during emergency situations such as severe allergies, ongoing treatments (eg, dialysis), rare serious conditions (eg, hemophilia), and medications dispensed at any Norwegian pharmacy [55]. Research shows that the most used function is the pharmaceutical tracking tool as it provides up-to-date medication information without additional manual data-entry requirements from physicians [56,57].

In 2013, the Directorate of Health recommended the integration of all eHealth and developed the initiative *One Patient–One Record* (*Én innbygger–Én journal*) [58,59]. In 2019, a US $296 million contract was signed with an American EHR company (Epic Systems) to eventually function as the nation’s sole HIT supplier [13,60]. The pilot program *Health Platform* (*Helseplattformen*) is scheduled to launch during the spring of 2022 in Central Norway, 1 of the 4 Regional Health Authorities [61]. Current studies indicate optimistic expectations mixed with concern as protected health information will eventually be exchanged across administrative, geographical, and institutional boundaries [62]. Regional governments created *consensus groups* comprised of health care professionals from >80 municipalities that are involved in software configuration and design [63]. After implementation, community physicians and analysts will continue to optimize the functionality for regional and practice needs, whereas Epic Systems will be involved to a lesser extent [13].

## Methods

### Participants and Setting

This study was conducted at the University of Kansas Medical Center (KUMC) in Kansas City, Kansas, and at the Akershus University Hospital in the Lørenskog municipality outside of Oslo. Bed capacity at each hospital was approximately 1000 beds [58,59]. Recruitment emails were sent to physicians involved in acute care at these facilities. In the United States, participants were board-certified EM physicians, whereas in Norway, participants were surgeons who provide services within the *akuttmottak*. A total of 12 interviews were conducted, 6 (50%) at each location. Average conversation lengths were 39.1 (SD 15.8) minutes.

### Data Collection

Data collection included face-to-face semistructured interviews and environmental observations. This was possible by conducting each interview on site at the hospitals. Participants were willing to show the typical documentation and clinical workflow to the interviewer (GG). This was essential when collecting Norwegian data, as the interviewer had no previous first-hand experience with this health care system. This provided context when participants referred to specifics of the EHR. Without this background the contextual understanding of participants’ answers would have been severely limited. All the interviews were conducted in English, as all the participants were proficient in this language. Conversations were audio-recorded on a passcode-protected device and then transcribed for further analysis. Privacy was retained by deidentifying the participants.

After obtaining written informed consent, standardized questions were used to obtain the following information from each participant: (1) demographics, (2) cultural and individual values, (3) individual comfort with general technology, (4) previous record experiences (electronic or paper), (5) observations of colleagues regarding EHR use, (6) individual attitudes toward EHR at current facility, (7) perceived usability (intuitive interfaces, software functionality, interoperability, workflow efficiencies, and centralized data repository), and (8) how the technology has shaped individual practice.

Follow-up questions varied based on individual responses. Participants were also asked about their knowledge, opinions, or questions regarding the other cohort’s electronic health care infrastructure. Natural conversation flow permitted additional discussion, allowing deeper exploration of ideas as they appeared organically. Additional questions developed throughout data collection were based on previous participant answers and cumulative observations. For example, US interviews were completed first and the responses involved specific negative consequences of the EHR without prompting. If these topics were never mentioned by the Norwegians, the interviewer inquired about them directly at the end of the discussion.

To conclude each interview, participants were asked if they had specific questions for the physicians in the other country. Following data collection, questions and answers were distributed to participants in addition to the background information on each country’s health care system. This allowed deeper understanding of individual perceptions while generating rich discussion. In addition, participants in Norway were explicitly asked about *Kjernejouralen* use. This study was reviewed by and received institutional review board approval from KUMC while abiding by the General Data Protection Regulation.

### Analysis

The US interviews were completed first, followed by interviews in Norway. Using grounded theory, themes emerged and evolved throughout the entire data collection process. As no single method captured the complexities of these data, analysis triangulation was necessary. First, transcripts underwent numerous thematic analyses to identify patterns between the cohorts. This was the initial formal approach to derive meaning from the vast and rich collected data. Similar to grounded theory, this exploratory methodology allows continuous hypothesis development throughout analysis progression. Narrative analysis was conducted to provide further insight into the mindset, perspectives, and attitudes toward EHRs. In addition, direct quotes were used to support the findings and may help the reader appreciate the nuances of the social context and emotion.

Early in the analysis process, 2 broad themes were identified—*perceived EHR benefits* and *perceived EHR disadvantages*. To gain deeper understanding of the data, interpretive phenomenological analysis and simple content analysis were used. Both methods aid in succinctly summarizing concepts based on individual experiences while providing some quantitative comparison. These techniques paired with the NVivo software (QSR International) helped to distinguish conceptual patterns between the cohorts, and ultimately resulted in the construction of the following 4 main code groups: *US perceived EHR benefits, Norway perceived EHR benefits, US perceived EHR disadvantages, and Norway perceived EHR disadvantages*.

**Figure 1:**
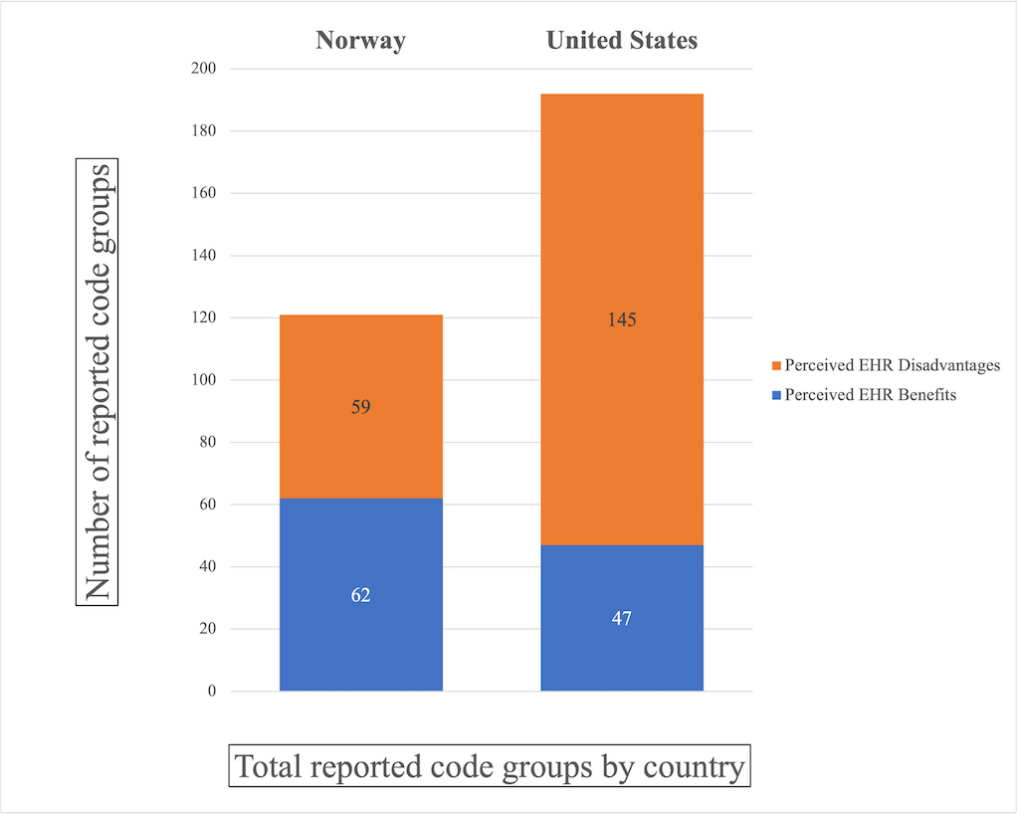
Total number of reported code groups by country. EHR: electronic health record.

Transcriptions were analyzed using the NVivo software (QSR International). As the perceived EHR benefits or disadvantages were found within the text, they were assigned to 1 of the 4 code groups based on context and cohort. The NVivo word frequency and query search functions were used to generate categories within the encoded text to enrich the results. The software allowed searches to include exact word matches, stemmed words, and synonyms. The search criteria details are presented in Textboxes 1 and 2. Identical queries regarding perceived EHR benefits and disadvantages were conducted for both cohorts. Query results were analyzed and refined to ensure that the terms were not taken out of context. The total number of results for each group was tabulated and compared. This is displayed in Figure 1. Comparing the categorical patterns provides concrete examples of varying priorities, opinions, and perspectives from the 2 cohorts. In addition, it examines the advantages and flaws of HIT implementation within each health care system.

**Categories of perceived electronic health record benefits.**

**Category and search criteria (include exact matches, stemmed words, and synonyms)**

- Patient safety and improved care: *safety*, *benefit*, *care*, *improve*, *alert*, *allergy*, *interaction*, *medication*, *automated*, *error*, *writing*, *legible*, and *mistakes*
- Access to useful clinical information: *accessibility*, *information*, *view*, *records*, *journal*, *chart*, *review*, *report*, *previous*, *tracking*, *results*, *history*, *important*, *critical*, *clinical*, *diagnosis*, *remote*, *exchange*, *facility*, *interoperable*, *capability*, *cloud*, *electronic*, *time*, and *speed*
- Data organization: organization, *sort*, *filter*, *search*, *usability*, *function*, *central*, *record*, *history*, *chart*, *journal*, *ease*, *efficient*, and *review*
- Enhanced communication: *communication*, *interaction*, *order*, *results*, *review*, *chart*, *record*, *journal*, *information*, *patient*, *encounter*, and *clarification*

**Categories of perceived electronic health record disadvantages.**

**Category and search criteria (include exact matches, stemmed words, and synonyms)**

- Excessive or irrelevant data: *excess*, *irrelevant*, *overload*, *quantity*, *redundant*, *limit*, *volume*, *amount*, *organize*, *filter*, *lost*, *search*, *data*, *clinical*, *benefit*, and *documentation*
- Poor interoperability: *interoperability*, *access*, *view*, *restrict*, *facility*, *exchange*, *data*, *information*, *chart*, *journal*, *record*, *outside*, *cloud*, *capability*, *hospital*, and *clinic*
- Increased workload: *work*, *workload*, *time*, *hour*, *administrative*, *requirement*, *documentation*, *efficient*, *amount*, *burden*, *click*, *task*, and *clerical*
- Software complexities: *software*, *complex*, *interface*, *intuitive*, *user*, *difficult*, *friendly*, *usability*, *navigate*, *understand*, *function*, *options*, *programs*, *system*, *load*, *slow*, *lag*, *ease*, *options*, *orders*, *run*, and *technology*
- Hardware malfunctions: *hardware*, *malfunction*, *crash*, *process*, *failure*, *update*, *IT*, *program*, *develop*, *technology*, *support*, *computer*, *device*, *speed*, and *paper*
- Financial influence: *financial*, *money*, *reimbursement*, *billing*, *profit*, *cost*, *incentive*, *code*, *dollars*, *relative value unit or RVU*, *regulation*, *mandate*, *clinical*, *value*, *price*, *payment*, and *business*

This process was conducted by the interviewer for retained consistency while considering abstract factors including nonverbal communication, clinical environment, and cultural norms. This technique was repeatedly used to explore topic relationships, consider causality, and help find thematic saturation within the populations.

## Results

### Overview

All the participants described both pros and cons of their experience with the EHRs and both groups agreed that modern medicine is heavily dependent on this technology. In general, Norwegian physicians had a slight propensity to report benefits (62 total perceived benefits reported) compared with disadvantages (59 total perceived disadvantages reported). In contrast, the American cohort frequently expressed unfavorable perceptions, reporting 145 total perceived disadvantages and only 47 total perceived benefits. These results are summarized in Figure 1.

### Perceived Benefits

Access to relevant patient information was the most commonly reported benefit in both countries. This included viewing previous diagnostic studies, clinical notes, and laboratory results. The Norwegian physicians were 1.7 times more likely to refer to these specific benefits (Norway [n=33] vs United States [n=20]). When American physicians mentioned this tool, they often also noted significant limitations owing to poor interoperability between competing HIT supply companies and health care facilities. A commonly perceived positive EHR outcome in both cohorts was improved patient safety. The results were moderately comparable between the 2 countries with American physicians referencing patient safety 20 times and Norwegian physicians referencing patient safety 15 times. An example that was frequently mentioned by participants was the automated alerts about patient allergies or drug–drug interactions. Many also believed that it has decreased unnecessary errors caused by illegible handwriting.

### Perceived Disadvantages

In general, there was a much broader range of topics related to perceived disadvantages when compared with benefits. The belief that EHRs have increased physicians’ workload was common to both cohorts. However, this was mentioned 2.6 times more frequently by the Americans (United States [n=40] vs Norway [n=15]). The most reported disadvantage was how increased clerical work detracted from efficiency. American physicians also discussed that they believe the required documentation has minimal, if any, clinical utility.

In Norway, the 2 most frequently discussed disadvantages of EHRs included software complexities (Norway [n=36] vs United States [n=25]) and hardware malfunctions (Norway [n=15] vs United States [n=7]). Every other disadvantage category was more common among the US cohort. In addition to increased workload, other categories included excessive and irrelevant data (United States [n=25] vs Norway [n=12]) and poor interoperability (United States [n=34] vs Norway [n=14]). The most significant difference between the cohorts was regarding the financial influence of the EHRs (United States [n=37] vs Norway [n=6]). Each American physician expressed without prompting that the primary purpose of EHRs within the United States is for billing rather than to improve patient care. This was often attributed to competing business models among HIT suppliers, insurance companies, and hospital administrations.

One disadvantage exclusive to the American cohort involved the legal implications of the EHRs. The interviewer never initiated this topic, yet it was brought up by half of the American participants. They strongly believed that the normalization of defensive medicine is a result of the society’s legal climate. Despite possessing adequate medical training and clinical judgment, clinicians often feel compelled to order extensive workups to protect themselves from future prosecution. In addition, these physicians mentioned that redundant testing is routinely performed because of limited HIE among surrounding health care facilities.

### Additional Observations

An interesting observation shared by both the cohorts was that their older colleagues expressed higher levels of EHR-related frustration. This was mentioned by 9 among all 12 participants—6 Norwegians and 3 Americans. These 9 individuals self-reported that they felt proficient in using technology but did not believe it influenced their own opinion of EHRs.

In addition, both Norwegians and Americans believed that the rapid processing speeds of personal devices may contribute to unrealistic EHR performance expectations. Many realized that top information technology developers are recruited to sectors outside of health care; however, they believed that usability would improve if companies such as Apple or Google developed the software:

It’s very hard to keep up with ever-changing new technology. As you get older you don’t have the stamina. Programs may also seem frustrating because they don’t run as quickly as most of our personal devices.

Norway, participant 4

At the start of each interview, the physicians were asked how cultural values influenced personal beliefs or medical practice, as other studies have described health care systems as a reflection of national ideals [26]. This question was intended to highlight nuanced variables that exist when comparing dissimilar populations. Unsurprisingly, the participant responses revealed differing values between the countries. Responses were not superior or inferior, just different. When describing how cultural values influence their current practice of medicine, the American participants used words such as *help*, *kind*, and *caring*. Common Norwegian terms included *open-minded*, *equality*, and *empathy*. Although these results have limited application in determining EHR satisfaction, it reinforces the importance of cultural context when developing solutions for specific populations.

## Discussion

Adding quantitative values to our qualitative analysis creates an overt visualization of the differences between EHR users in both countries. We have provided a more comprehensive exploration of the influencing factors.

### Clerical Burden and Reimbursement

Increased administrative tasks that yield minimal patient benefit created frustration for all physicians; however, it was significantly higher among US participants. In Norway, physicians must include appropriate diagnosis or procedure codes for hospital reimbursement using the Diagnosis Related Groups system, which includes approximately 980 codes [64]. These codes generate approximately 50% of the hospital revenue, with the remaining financed from fixed government payments [65]. Norwegians are skeptical of potential changes following the national implementation of Epic Systems. The participants voiced concern regarding slowly evolving into an American health care model. Some Norwegian participants first noticed this shift after hospital reimbursement became partially integrated with diagnosis codes:

With new public management reform within the last 30 years, we have also noticed health care has changed to suit their needs. Most Norwegian physicians are attentive and oppose this. We also have the union (Norwegian Medical Association) who oppose it. It isn’t in our immediate power to change those things and they must come from a higher level.

Norway, participant 6

American reimbursement is complex owing to a multi-payer system that includes government agencies, insurance companies, health maintenance organizations, employers, and individual patients [66]. Although many countries use the International Classification of Disease, the United States is one of the few countries that use it for both diagnosis and billing, while including more than 90,000 codes [67]. Compared with Norway, the United States uses the entire medical record for reimbursement. The billing *level* is determined by the quantity of the documented elements within each note section (ie, history of present illness, review of systems, and physical exam) with more elements correlating with higher billing levels, resulting in increased reimbursement [68]. Physicians must also filter through long, redundant, and confusing lists of diagnoses to choose the most detailed option [24]. Another form of reimbursement, relative value units, is also extracted from the EHR. These are based on >8000 procedure codes extrapolated to measure physician productivity, which are then used to determine department or individual reimbursement [66,69]; 1 physician shared that for the last 2 years, 25% of their salaries depended on the individual relative value units generated:

Institutions now look at emergency departments as revenue generators. We cost society more and in the end the patient loses directly and indirectly.

United States, participant 3

American documentation tends to be 4 times longer than that of other countries, without offering any additional clinical information [48]. The position of *medical scribes* (nonclinical personnel who are trained to provide documentation assistance and workflow support) was created as a possible solution to this problem. Research demonstrates that scribes are valued team members and improve provider satisfaction [70]. This sentiment was echoed by American physicians, whereas Norwegians were unfamiliar with this occupation. Gardner et al [1] showed great variety of scribe use among American specialties, with the highest use among EM physicians. This study also found that working with scribes reduced the odds of burnout by approximately 40%. They hypothesized that it was not higher because scribes are not qualified to complete certain time-consuming but physician-specific electronic tasks (eg, medication orders and in-basket management) [1]:

We are so opposed to these tasks that steal time we could otherwise use for clinical work. Don’t you think that having a scribe is just a waste of resources? Do individual physicians actually generate enough data on a single patient that they need a scribe to help complete the documentation?

Norway, participant 6

### Burnout

Multiple studies within the United States indicate that HIT creates undue physician burden and there is considerable correlation between high EHR frustration and burnout [1,4,7,24,43,71-76]. The 2018 National Physician Poll produced powerful data regarding how this technology affects American physicians. Only 8% of participants believed that the primary purpose of documentation is clinical, whereas 71% agreed that it significantly contributes to burnout [77]. Our study supports this argument, as many American physicians cited EHRs as a significant cause of burnout. However, these individuals clarified that it is only a single contributor to a complex and multifactorial issue:

I don’t necessarily think the electronic aspect of EHRs are what makes them so frustrating, but rather the need of documenting in excess. When you have to do such complicated things against your will and without patient benefit, it adds to burnout rates.

United States, participant 6

In Norway, burnout was never mentioned spontaneously and eventually the interviewer was required to ask about it explicitly. Norwegians attributed burnout to perceived job demands, societal expectations, and degree of colleague support, which is consistent with other Norwegian studies [78-80]. EHRs were never mentioned as a source of burnout. Since 1993, Norway has conducted extensive research aimed at improving physicians’ health, working conditions, and quality of life [81]. Despite burnout being less prevalent, Norway has established proactive prevention initiatives. An example is a self-referral physician counseling program and treatment facility (Villa Sana) designed to enhance coping skills and reduce emotional exhaustion [82,83].

West et al [76] considered factors that contribute to physician burnout from a global perspective. In doing so, they highlighted a previous Norwegian study that found no significant difference in burnout between physicians and other professions [84]. However, in the same study, there was a significantly increased prevalence of burnout in the United States even after adjusting for work hours and other factors [76]. Another recent US study identified systemic issues contributing to EM physician burnout. Factors include EHR limitations, long work hours, substantial educational debt, intense clinical practice, high risk of litigation, circadian rhythm disruption, chronic fatigue, blame, and isolation as a result of poor outcomes, all within the confines of an environment with zero tolerance for mistakes [85]. Our study offers informal evidence that EHRs increase burnout risk in the United States but appear noncontributory in Norway.

### Core Journal (Kjernejournalen)

Of the 6 Norwegian physicians, 5 used the *Kjernejournalen* at least multiple times per week. Most information required initial manual entry, which has created additional tasks for providers. Some participants also attributed slow processing speeds as a reason for their limited use. However, the *Kjernejournalen* software provides a function that was highly favored by all the Norwegian physicians in this study—the pharmaceutical tracking function. The *Kjernejournalen* connects with all the pharmacies in the country and updates automatically as prescriptions are filled [55,56]. This tool was favored as it provides useful information without increasing data entry responsibilities. When asked if the Core Journal has affected their medical practice, the first participant provided the following response:

I would say that one way is you can now see what is prescribed and if it has been collected. It is a more secure way of finding out what patients are really taking.

Norway, participant 1

Overall, there were mixed feelings about the software among Norwegian physicians. In contrast, all the American physicians expressed their desire for something similar upon learning about the Core Journal. They were also interested in the pharmaceutical tracking function, specifically for narcotic medications. Of the 50 states, all except one (Missouri) have state-wide tracking software; however, communication between programs is limited [86]. In addition, the American cohort at the KUMC faces the unique challenge of working within a facility that is geographically located on the Kansas–Missouri state-line border.

### Interoperability

In-depth conversation regarding EHR interoperability capabilities revealed significantly different experiences between the EDs in the 2 countries. In Norway, specialty care is confined to hospitals and allows EM physicians to easily view specialist or inpatient notes. However, primary care facilities are part of the private sector and use different EHRs. Hospital and primary physicians alike are able to access the Core Journal, which provides information regarding critical diagnosis and current prescriptions [55]. However, Norwegian participants indicated that emergency care was never impeded because of the inability to access primary care clinic notes. Instead, their frustration occurred when requesting imaging from distant facilities. Both cohorts reported needing outside records and imaging occasionally. All the physicians found this task to be annoying and time-consuming. In Norway, all radiologic studies can be electronically exchanged among health care systems throughout the country and sometimes require several phone calls. It was reported that this can take up to 20 minutes but is typically completed more quickly. American physicians noted that they can occasionally view outside imaging. However, this is often not available and scans have to be repeated.

Patients in the United States often receive both primary or specialist care in an outpatient clinic setting. Providers have limited access to patient information at the point of care if health care facilities use different HIT suppliers [12,87]. Over the past decade, laws have been passed with the goal of improving interoperability, but definitive legal parameters are yet to be firmly established [12,45,46]. HIE configuration decisions are typically dependent on the competing vendors and participating health care systems, with both parties having significant effect on user accessibility [48]. Vendors have capitalized on developing exchange capabilities as a product selling point [46]. Subsequently, there have been calls for stronger legislative regulation to improve transparency across health care facilities [41,45,46].

Although individual EHR suppliers have improved interoperability, substantial limitations persist [19]. For example, American physicians in this study discussed the Care Everywhere platform within Epic Systems that grants access to most outpatient documentation and laboratory results from another large hospital within Kansas City. However, this tool still omits numerous facilities and hospitals. US participants reported that electronic exchanges between unaffiliated health care facilities are either impossible or extremely cumbersome and time-consuming. A participant described the process used to request outside medical records and said that it could take hours to days to receive a fax that potentially contains critical information. Knowing that the information will not be available within their own shift, this participant typically makes these requests to benefit colleagues who are taking over patient care. American physicians also believed that redundant diagnostic tests are a direct result of limited interoperability that increases both patient risk and national health care expenses:

We repeat so many x-rays, labs, and scans just because we can’t see what was done a day ago. There are deficits in care due to poor EHR interoperability. Today, in this emergency department, there will be an issue because they [outside EHRs] don’t communicate.

United States, participant 6

The VA is a government-run national health care system that internally developed its own EHR software known as Veterans Information Systems and Technology Architecture [41]. Each US participant who mentioned past VA experiences recalled positive experience with this EHR. Although the participants described the software’s interface as cumbersome and rudimental, all of them commented about how it allowed them to provide more comprehensive care because of the ability to access all the pertinent information from any VA facility. Despite the recent contract with Cerner, it will likely take longer than 10 years to finalize the implementation of this software as the sole HIT supplier to all VA facilities [41,49].

### Legal Considerations

Another burden unique to Americans is the extensive documentation for legal protection. A recent study showed that approximately 51% of EM physicians in the United States will be sued during their career despite appropriate medical management [88]. This was foreign to Norwegians who rely on the Norwegian Medical Association (NMA) for legal counsel and protection [89]. NMA also functions as a professional society and labor union that annually negotiates with the government on behalf of physicians regarding fair working conditions, compensation, and leave-time [89]. Nearly all Norwegian physicians are NMA members, whereas only 11.4% of American health care providers are unionized [90]. Explanations for low involvement include convoluted multi-payer systems, restrictive federal and state laws, and social stigma [90-92].

Defensive medicine is a normalized practice within US medicine. American EM physicians face approximately a 7.5% annual risk of litigation [93]. Consequently, excessive documentation becomes an essential burden to protect oneself from potential legal ramifications. This liability heavily influences medical decision-making, resulting in excessive workups and hospital admissions. A study of 824 physicians in the United States found that 93% of them reported regular practice of defensive medicine [94]. Of those, more than half of the EM physicians reported using computed tomography, magnetic resonance imaging, or radiography that was not clinically necessary [94].

Responses from the American participants correlated with these findings and many believed that improved interoperability between EHR systems could mitigate these practices while simultaneously decreasing physician litigation anxiety. American participants also noted numerous disadvantages associated with defensive medicine on a societal, patient, and health care provider perspectives; however, abandoning this practice puts the physician at an undeniable risk:

A lawyer can go through and subpoena every keystroke made from the moment you enter the record, what is done before completing the note, and if you changed anything. We are humans and will make mistakes. If you type something wrong, it can potentially be used against you to criticize your medical judgment. If I have a learner (i.e., scribe or resident) who wrote something wrong and I change or delete it, that may be held against me.

United States, participant 6

This is in stark contrast to the practices in Norway, where physicians pay a small percentage of their salaries to a collective pool within the NMA. If a patient is entitled to compensation, it comes from these funds. All Norwegian participants expressed that this was a fair and equitable process without many disadvantages, and one physician stated the following:

I am only concerned for malpractice because I am always concerned with doing the right thing for my patient. I am not concerned about repercussions for making a mistake. When something goes wrong, we are good at protecting each other and focusing on system errors, not personal ones.

Norway, participant 2

### Limitations and Future Implications

Our study has several limitations. Qualitative research restricts the use of formal statistical analysis as broad-ranging emotions reduce its reproducibility. These challenges were amplified by complex sociopolitical–technological variations. Generalizability is limited owing to the small sample size and single-center analysis in each country. Therefore, we can only extrapolate speculations to explain the results of this study. No definitive conclusions can be made regarding EHR user satisfaction between the 2 countries. Although this study specifically recruited EM physicians, future research may benefit from expanding to other specialties across multiple facilities. Despite the semistructured interviews having reproducibility limitations, this method was necessary to understand the health care infrastructure and nuances of daily practice within each location. New questions emerged as more information was gained. Although this approach creates inconsistencies, it permits flexibility that is otherwise impossible to achieve using alternative qualitative methods such as surveys. These humanistic interactions are both a strength and weakness of semistructured interviews. Objective metrics regarding usability and satisfaction are difficult to produce with countless independent variables. Nevertheless, this comparison provides rich insight.

Numerous potential factors that may contribute to poor EHR user experiences were identified during the first phase of data collection (American interviews). Much of this occurred without prompts from the interviewer (GG). If these factors did not come up organically in Norwegian physician interviews, the interviewer asked targeted questions pertaining to these topics with the intention of identifying similarities or differences. Although this does not alter the United States’ findings, it may artificially inflate Norwegian results regarding perceived EHR disadvantages.

A study by Tutty et al [14] described factors that may enhance EHR experiences and suggested that policy makers, software developers, HIT vendors, payers, health administrators, and users alike may be capable of contributing to collective improvements. They also identified administrative tasks that add to documentation burden, including extensive order entries, billing regulations, coding standards, quality improvement reporting, and system security [14]. As Colicchio et al [40] noted, it is important to consider that national EHRs may not provide the desired insight for future informatics research, as local configurations are customizable even when supplied by the same vendor. After the *Helseplattformen* is implemented in Norway, prospective longitudinal studies measuring similar outcomes may produce additional meaningful information. This novel investigation suggests a framework for theoretical EHR optimization on a global scale. Although the results of this study are not entirely generalizable, it provides a foothold for future research and may stimulate innovative HIT advancements. Additional studies that compare international experiences while considering social and political differences are needed to identify the components that most significantly influence user satisfaction.

### Conclusions

This qualitative study explores factors that influence EHR user satisfaction among practicing EM physicians in 2 countries. All the participants believed that this technology has increased their workload while simultaneously acknowledging their heavy reliance on it. They agreed that EHRs are here to stay. The results show that both American and Norwegian physicians experience frustration with EHR, but overall, the United States cohort had significantly more complaints. Participant-driven conversations revealed that each country had moderately differing sources of frustration. Norwegian complaints revolved around intrinsic technical issues. Strategies to mitigate these problems are currently underway as evidenced by the *Én Innbygger–Én Journal* and *Helseplattformen* initiative. Americans harshly criticized the *business of medicine* that they felt was manifested in every facet of HIT implementation. These findings enhance the theory that policies and administration may influence usability to a greater degree than technology itself [9,14,24,26,95].

Use of in-depth, semistructured interviews permitted a deeper understanding of both health care systems. This knowledge was subsequently integrated throughout data analysis and interpretation. The development and use of EHRs is influenced by lawmakers, payers, companies, and regulatory entities. Decisions made by those who are not primary users have a profound impact on the practice of those who use this technology daily. Both countries in this study are currently undergoing significant changes. Norway is poised to make a complete national overhaul of their EHR, and the United States is struggling to reform a vast, expensive, and inefficient health system. If HIT is to be optimized on a global scale, the elements highlighted in this study should be considered when establishing policy, strategy, and vision for the future.

## Abbreviations

ED: emergency department
EHR: electronic health record
EM: emergency medicine
HIE: health information exchange
HIT: health information technology
KU: University of Kansas
KUMC: University of Kansas Medical Center
NMA: Norwegian Medical Association
VA: US Department of Veterans Affairs

## References

[1] Gardner RL, Cooper E, Haskell J, Harris DA, Poplau S, Kroth PJ, Linzer M (2019). Physician stress and burnout: the impact of health information technology. J Am Med Inform Assoc.

[2] Denton CA, Soni HC, Kannampallil TG, Serrichio A, Shapiro JS, Traub SJ, Patel VL (2018). Emergency physicians' perceived influence of ehr use on clinical workflow and performance metrics. Appl Clin Inform.

[3] Perry JJ, Sutherland J, Symington C, Dorland K, Mansour M, Stiell IG (2014). Assessment of the impact on time to complete medical record using an electronic medical record versus a paper record on emergency department patients: a study. Emerg Med J.

[4] Hill RG, Sears LM, Melanson SW (2013). 4000 clicks: a productivity analysis of electronic medical records in a community hospital ED. Am J Emerg Med.

[5] Neri PM, Redden L, Poole S, Pozner CN, Horsky J, Raja AS, Poon E, Schiff G, Landman A (2015). Emergency medicine resident physicians' perceptions of electronic documentation and workflow: a mixed methods study. Appl Clin Inform.

[6] Park SY, Lee SY, Chen Y (2012). The effects of EMR deployment on doctors' work practices: a qualitative study in the emergency department of a teaching hospital. Int J Med Inform.

[7] Hecht J (2019). The future of electronic health records. Nature.

[8] Arndt BG, Beasley JW, Watkinson MD, Temte JL, Tuan W, Sinsky CA, Gilchrist VJ (2017). Tethered to the EHR: primary care physician workload assessment using ehr event log data and time-motion observations. Ann Fam Med.

[9] Friedberg MW, Chen PG, Van Busum KR, Aunon F, Pham C, Caloyeras J, Mattke S, Pitchforth E, Quigley DD, Brook RH, Crosson FJ, Tutty M (2014). Factors affecting physician professional satisfaction and their implications for patient care, health systems, and health policy. Rand Health Q.

[10] Sinsky C, Colligan L, Li L, Prgomet M, Reynolds S, Goeders L, Westbrook J, Tutty M, Blike G (2016). Allocation of physician time in ambulatory practice: a time and motion study in 4 specialties. Ann Intern Med.

[11] Khairat S, Coleman C, Newlin T, Rand V, Ottmar P, Bice T, Carson SS (2019). A mixed-methods evaluation framework for electronic health records usability studies. J Biomed Inform.

[12] Office of the National Coordinator for Health Information Technology (ONC) (2018). 2018 Report to Congress. Department of Health & Human Services (HHS).

[13] Hertzum M, Ellingsen G (2019). The implementation of an electronic health record: comparing preparations for epic in Norway with experiences from the UK and Denmark. Int J Med Inform.

[14] Tutty MA, Carlasare LE, Lloyd S, Sinsky CA (2019). The complex case of EHRs: examining the factors impacting the EHR user experience. J Am Med Inform Assoc.

[15] Garavand A, Mohseni M, Asadi H, Etemadi M, Moradi-Joo M, Moosavi A (2016). Factors influencing the adoption of health information technologies: a systematic review. Electron Physician.

[16] Evans RS (2016). Electronic health records: then, now, and in the future. Yearb Med Inform.

[17] Kaipio J, Lääveri T, Hyppönen H, Vainiomäki S, Reponen J, Kushniruk A, Borycki E, Vänskä J (2017). Usability problems do not heal by themselves: National Survey on physicians' experiences with EHRs in Finland. Int J Med Inform.

[18] Jha AK, Doolan D, Grandt D, Scott T, Bates DW (2008). The use of health information technology in seven nations. Int J Med Inform.

[19] Payne TH, Lovis C, Gutteridge C, Pagliari C, Natarajan S, Yong C, Zhao L (2019). Status of health information exchange: a comparison of six countries. J Glob Health.

[20] Teixeira JG, Pinho NF, Patrício L (2019). Bringing service design to the development of health information systems: the case of the Portuguese national electronic health record. Int J Med Inform.

[21] Powell AC, Ludhar JK, Ostrovsky Y (2017). Electronic health record use in an affluent region in India: findings from a survey of Chandigarh hospitals. Int J Med Inform.

[22] Salleh MI, Abdullah R, Zakaria N (2021). Evaluating the effects of electronic health records system adoption on the performance of Malaysian health care providers. BMC Med Inform Decis Mak.

[23] Kushniruk AW, Bates DW, Bainbridge M, Househ MS, Borycki EM (2013). National efforts to improve health information system safety in Canada, the United States of America and England. Int J Med Inform.

[24] Downing NL, Bates DW, Longhurst CA (2018). Physician burnout in the electronic health record era: are we ignoring the real cause?. Ann Intern Med.

[25] Faber S, van Geenhuizen M, de Reuver M (2017). eHealth adoption factors in medical hospitals: a focus on the Netherlands. Int J Med Inform.

[26] Whyle E, Olivier J (2020). Social values and health systems in health policy and systems research: a mixed-method systematic review and evidence map. Health Policy Plan.

[27] Halamka JD, Tripathi M (2017). The HITECH era in retrospect. N Engl J Med.

[28] Sawyer NT (2017). Why the EMTALA mandate for emergency care does not equal healthcare "Coverage". West J Emerg Med.

[29] Zibulewsky J (2001). The Emergency Medical Treatment and Active Labor Act (EMTALA): what it is and what it means for physicians. Proc (Bayl Univ Med Cent).

[30] EMTALA fact sheet. American College of Emergency Physicians.

[31] Shenvi C, Beise K, Tintinalli J (2013). 30 años de programas de residencia en Medicina de Urgencias y Emergencias en Estados Unidos. Emergencias.

[32] Augustine J (2019). Latest data reveal the ED's role as hospital admission gatekeeper. American College of Emergency Physicians.

[33] Gindi RM, Black LI, Cohen RA (2016). Reasons for Emergency Room Use Among U.S. Adults Aged 18-64: National Health Interview Survey, 2013 and 2014. Natl Health Stat Report.

[34] (2020). Consumer expenditure survey - Health insurance. U.S. Bureau of Labor Statistics.

[35] Schoen C, Osborn R, Squires D, Doty MM, Pierson R, Applebaum S (2010). How health insurance design affects access to care and costs, by income, in eleven countries. Health Aff (Millwood).

[36] Capp R, Rooks SP, Wiler JL, Zane RD, Ginde AA (2014). National study of health insurance type and reasons for emergency department use. J Gen Intern Med.

[37] Richman IB, Clark S, Sullivan AF, Camargo CA (2007). National study of the relation of primary care shortages to emergency department utilization. Acad Emerg Med.

[38] Washington V, DeSalvo K, Mostashari F, Blumenthal D (2017). The HITECH era and the path forward. N Engl J Med.

[39] (2020). Meaningful use: Electronic Health Record (EHR) incentive programs. American Medical Association.

[40] Colicchio TK, Cimino JJ, Del Fiol G (2019). Unintended consequences of nationwide electronic health record adoption: challenges and opportunities in the post-meaningful use era. J Med Internet Res.

[41] Reisman M (2017). EHRs: the challenge of making electronic data usable and interoperable. P T.

[42] Ratwani RM, Fairbanks Rollin J, Hettinger A Zachary, Benda Natalie C (2015). Electronic health record usability: analysis of the user-centered design processes of eleven electronic health record vendors. J Am Med Inform Assoc.

[43] Schulte F, Fry E (2019). Death by 1,000 clicks: where electronic health records went wrong. Kaiser Health News.

[44] Non-federal acute care hospital electronic health record adoption. Health IT Dashboard.

[45] Black JR, Hulkower RL, Ramanathan T (2018). Health Information Blocking: Responses Under the 21st Century Cures Act. Public Health Rep.

[46] Adler-Milstein J, Pfeifer E (2017). Information blocking: is it occurring and what policy strategies can address it?. Milbank Q.

[47] (2018). Health information technology: certification and interoperability enhancements. Department of Health and Human.

[48] Downing NL, Adler-Milstein J, Palma JP, Lane S, Eisenberg M, Sharp C, Longhurst CA, Northern California HIE Collaborative (2017). Health information exchange policies of 11 diverse health systems and the associated impact on volume of exchange. J Am Med Inform Assoc.

[49] (2018). Cerner, VA to provide Veterans with seamless care. Federal Update.

[50] Ringard AS, Sagan A, Sperre Saunes I, Lindahl AK (2013). Norway: health system review. Health Syst Transit.

[51] (2020). What is core journal?. Norwegian Health Network.

[52] Galletta G, Løvstakken K (2020). Emergency medicine in Norway: the road to specialty recognition. J Am Coll Emerg Physicians Open.

[53] Mentzoni I, Bogstrand ST, Faiz KW (2019). Emergency department crowding and length of stay before and after an increased catchment area. BMC Health Serv Res.

[54] Arnesen EN (2017). E-Health in Norway. E-health in Hospital Care in 2017.

[55] Arnesen EN, Larsen BA (2014). Alert information in the Norwegian Summary Care Record. Tidsskr Nor Laegeforen.

[56] Dyb K, Warth LL (2018). The Norwegian National Summary Care Record: a qualitative analysis of doctors' use of and trust in shared patient information. BMC Health Serv Res.

[57] Warth L, Dyb K (2017). A qualitative study of the implementation and use of a national information system. International Association for Development of the Information Society (IADIS).

[58] (2013). One inhabitant- one journal. Ministry of Health and Care Services.

[59] Green A (2018). EMR/EHR developments in the Nordics - White paper. Signify Research Knowledge Centre.

[60] Onarheim K (2019). Helseplattformen- one health record across all levels of care- a ground-breaking project in Central Norway. Proceedings of the European Conference and Exhibition.

[61] (2019). Helseplattformen AS. Helseplattformen.

[62] Melby LM, Andreassen HK, Torsvik T, Ellingsen G, Severinsen GH, Silsand L, Ekeland AG, Saadatfard O, Pedersen R (2019). Ambivalently awaiting: Norwegian general practitioners' expectations towards a cross-institutional electronic health record. Proceedings of the Linköping Electronic Conference.

[63] (2020). The project. Helseplattformen.

[64] (2020). Effort-driven financing (ISF) - regulations. Helsedirektoratet.

[65] Tikkanen R, Osborn R, Mossialos E, Djordjevic A, Wharton GA Norway. The Commonwealth Fund.

[66] Blome A, Yu D, Lu X, Schreyer KE (2020). Pitfalls of Extensive Documentation in the Emergency Department. Ochsner J.

[67] (2019). International Classification of Diseases Eleventh Revision (ICD-11). National Committee on Vital and Health Statistics (NCVHS).

[68] Levinson DR (2014). Improper payments for evaluation and management services cost medicare billions in 2010. Department of Health and Human Services.

[69] Proctor J Gauging emergency physician productivity: are RVUs the answer?. American College of Emergency Physicians.

[70] Gottlieb M, Palter J, Westrick J, Peksa GD (2021). Effect of medical scribes on throughput, revenue, and patient and provider satisfaction: a systematic review and meta-analysis. Ann Emerg Med.

[71] Tajirian T, Stergiopoulos V, Strudwick G, Sequeira L, Sanches M, Kemp J, Ramamoorthi K, Zhang T, Jankowicz D (2020). The influence of electronic health record use on physician burnout: cross-sectional survey. J Med Internet Res.

[72] Khairat S, Burke G, Archambault H, Schwartz T, Larson J, Ratwani R (2018). Perceived burden of EHRs on physicians at different stages of their career. Appl Clin Inform.

[73] Robertson SL, Robinson MD, Reid A (2017). Electronic Health Record Effects on Work-Life Balance and Burnout Within the I Population Collaborative. J Grad Med Educ.

[74] Shanafelt TD, Dyrbye LN, Sinsky C, Hasan O, Satele D, Sloan J, West CP (2016). Relationship between clerical burden and characteristics of the electronic environment with physician burnout and professional satisfaction. Mayo Clin Proc.

[75] Shanafelt TD, Noseworthy JH (2016). Executive leadership and physician well-being: nine organizational strategies to promote engagement and reduce burnout. Mayo Clin Proc.

[76] West CP, Dyrbye LN, Shanafelt TD (2018). Physician burnout: contributors, consequences and solutions. J Intern Med.

[77] (2018). How doctors feel about electronic health records: the Harris poll. Stanford Medicine.

[78] Mahmood JI, Grotmol KS, Tesli M, Moum T, Andreassen O, Tyssen R (2019). Life satisfaction in Norwegian medical doctors: a 15-year longitudinal study of work-related predictors. BMC Health Serv Res.

[79] Hertzberg TK, Skirbekk H, Tyssen R, Aasland OG, Rø KI (2016). The hospital doctor of today - still continuously on duty. Tidsskr Nor Laegeforen.

[80] Røvik JO, Tyssen R, Hem E, Gude T, Ekeberg O, Moum T, Vaglum P (2007). Job stress in young physicians with an emphasis on the work-home interface: a nine-year, nationwide and longitudinal study of its course and predictors. Ind Health.

[81] Tyssen R (2007). Health problems and the use of health services among physicians: a review article with particular emphasis on Norwegian studies. Ind Health.

[82] Rø KEI, Gude T, Aasland OG (2007). Does a self-referral counselling program reach doctors in need of help? A comparison with the general Norwegian doctor workforce. BMC Public Health.

[83] Isaksson Ro KE, Tyssen R, Gude T, Aasland OG (2012). Will sick leave after a counselling intervention prevent later burnout? A 3-year follow-up study of Norwegian doctors. Scand J Public Health.

[84] Langballe EM, Innstrand ST, Hagtvet KA, Falkum E, Gjerløw Aasland O (2009). The relationship between burnout and musculoskeletal pain in seven Norwegian occupational groups. Work.

[85] Stehman CR, Testo Z, Gershaw RS, Kellogg AR (2019). Burnout, drop out, suicide: physician loss in emergency medicine, part I. West J Emerg Med.

[86] Weber L (2019). Why Missouri’s the Last Holdout on a Statewide Rx Monitoring Program. Kaiser Health News.

[87] Kern LM, Seirup JK, Rajan M, Jawahar R, Stuard SS (2018). Fragmented ambulatory care and subsequent healthcare utilization among Medicare beneficiaries. Am J Manag Care.

[88] Guardado JR (2017). Medical liability claim frequency among U.S. physicians. American Medical Association.

[89] (2018). The role of the Norwegian Medical Association. Den Norske Legeforening.

[90] Howard D (2020). What should physicians consider prior to unionizing?. AMA J Ethics.

[91] Adams MA, Allen JI (2018). Understanding the Legal and Regulatory Framework Governing Physician Collective Bargaining. Am J Gastroenterol.

[92] Choudhry S, Brennan TA (2001). Collective bargaining by physicians--labor law, antitrust law, and organized medicine. N Engl J Med.

[93] Ferguson B, Geralds J, Petrey J, Huecker M (2018). Malpractice in emergency medicine-a review of risk and mitigation practices for the emergency medicine provider. J Emerg Med.

[94] Studdert DM, Mello MM, Sage WM, DesRoches CM, Peugh J, Zapert K, Brennan TA (2005). Defensive medicine among high-risk specialist physicians in a volatile malpractice environment. J Am Med Assoc.

[95] Middleton B, Bloomrosen M, Dente MA, Hashmat B, Koppel R, Overhage JM, Payne TH, Rosenbloom ST, Weaver C, Zhang J, American Medical Informatics Association (2013). Enhancing patient safety and quality of care by improving the usability of electronic health record systems: recommendations from AMIA. J Am Med Inform Assoc.

